# 

**Published:** 1896-12-26

**Authors:** 


					CADBURY'S WW ?*. CADBURY'S
. OOCOA ^ ? Ifrn
? !x " The standard of highest purity ai present (S^> 'A
a ttalnable."?L ance t ?
COOOA
NO ALKALIES USED.
ULAsaotr -Western Lnflrmarx
No. 53o. - iVol. XXI.
Saturday.
Deo. 26th, 1896.
NOR WICH HOSfA.TAt ?"
PRICE TWOPENCE.
[Registered at the General Post Offioe aa a Newspaper for Per Annam, 10s. 6d., post free,
transmission in the United Kingdom and Abroad.] Monthly Parts, 6d.; post free, 8d.
Ditto per Annum, 8s. 6d.t post tree.

				

